# Targeted deletion of miR-182, an abundant retinal microRNA

**Published:** 2009-03-09

**Authors:** Zi-Bing Jin, Go Hirokawa, Le Gui, Rie Takahashi, Fumitaka Osakada, Yumiko Hiura, Masayo Takahashi, Osamu Yasuhara, Naoharu Iwai

**Affiliations:** 1 Laboratory for Retinal Regeneration, RIKEN Center for Developmental Biology, Kobe, Hyogo, Japan; 2 Department of Epidemiology, Research Institute, National Cardiovascular Center, Suita, Osaka, Japan; 3 Molecular Neuroscience Research Center, Shiga University of Medical Science, Otsu, Japan

## Abstract

**Purpose:**

MicroRNA-182 (miR-182) is expressed abundantly in the mammalian retina and is therefore thought to perform important roles for the retinal development and the function. To test this hypothesis, we generated miR-182 knockout mice.

**Methods:**

northern blotting was performed to confirm the robust expression of miR-182 in the eye. The precursor sequence of miR-182 was replaced by the neomycin resistance gene under the control of the phosphoglycerate kinase 1 promoter in a targeting construct. The targeting vector was linearized and transfected into embryonic stem (ES) cells. Recombinant ES clones were selected and injected into blastocysts to generate male chimeras. Heterozygous and homozygous mice were obtained after five generations of backcrossing and were confirmed using genotyping and northern blotting.

**Results:**

Heterozygous (+/−) and homozygous (−/−) knockout mice were morphologically normal, viable, and fertile. Immunohistochemical analysis of the miR-182–deficient retinas did not reveal any apparent structural abnormalities in the retinas. Consistently, global expression profiling using a repeated microarray did not identify significant fluctuations for potential target genes.

**Conclusions:**

We successfully generated miR-182 knockout mice and characterized the resulting miR-182–depleted retina. This is the first report describing the targeted deletion of a single miRNA that is highly expressed in the retina. The absence of significant transcriptional and phenotypic changes in miR-182–depleted retinas suggests that miR-182 is not a major determinant of retinal development or delamination. Further studies are required to elucidate any functional changes in the retina.

## Introduction

MicroRNAs (miRNAs) are a class of short, single-stranded RNA molecules that regulate gene expression [1-3]. In general, miRNA genes are transcribed to generate primary transcripts (pri-miRNAs) in the nucleus. Pri-miRNAs then are cropped by nuclear RNase into pre-miRNA hairpin precursors and exported into the cytoplasm. Cytoplasmic pre-miRNAs are then processed into mature miRNA molecules by Dicer. These mature molecules are able to bind to partially complementary sequences within the 3′ untranslated region (UTR) of target mRNAs. MicroRNAs have been computationally predicted to regulate more than one-third of human gene transcripts [4-6], and more than 500 miRNAs have been identified to date, a number that is rapidly increasing. Many lines of evidence have suggested critical roles for the miRNA system in various biologic processes, including development, cancer biology, and other pathologic conditions.

At least 78 miRNAs have been found to be preferentially or specifically expressed in the retina [7-9]. These miRNAs are suspected to play important roles in retinal cell differentiation, proliferation, development, and apoptosis by modulating gene expression profiles. Recently, several groups reported the retinal expression of a polycistronic miRNA cluster, which includes miR-182, miR-183, and miR-96 in the retina [9,10]. The expression levels of this phylogenetically conserved cluster of miRNA genes markedly increase from developmental stage postnatal day 1 (P1) to adulthood [9]. In a mouse model of retinitis pigmentosa, the expression levels of these miRNAs were significantly lower compared with results from wild-type mice [9,10]. Thus, the miR-182 gene cluster may play a critical role in retinal development and physiology.

Eliminating various miRNAs via a knockout (KO) approach in mice has revealed their essential roles in cardiac growth and development, the germinal center response, homeostasis, and immunity [11-14]. Damiani et al. reported that broad inactivation of miRNAs by removing Dicer from the retina leads to progressive and widespread structural abnormalities [15]. We attempted to elucidate the specific roles of miR-182 in retinal development by generating a miR-182 KO mouse line. Here, we report the results from our initial examination of the miR-182 KO mice.

## Methods

All animal experiments were conducted in accordance with the ARVO Statement for the Use of Animals in Ophthalmic and Vision Research and the guidelines for the care and use of experimental animals of the National Cardiovascular Center. This study was approved by the Committee of Animal Use of the National Cardiovascular Center.

### Northern blot analysis

Total RNA was isolated from mouse tissues using the TRIzol reagent (Invitrogen, Carlsbad, CA), and northern blotting was performed as described previously [16]. Briefly, 10 μg of total RNA was separated using a 15% denaturing polyacrylamide gel and transferred to a Zetaprobe membrane (BioRad, Hercules, CA). Oligonucleotide probes specific for miR-96, miR-182, and miR-183 (IDT Technologies, Coralville, IA) were labeled with [α-^32^P]dATP. Hybridization was performed overnight at 42 °C (miR-96) or 35 °C (miR-182 and miR-183), and the signals were detected using a BAS2500 image analyzer (Fuji Photo Film, Tokyo, Japan).

### MiR-182-specific in situ hybridization

Eyes at embryonic day (E) 14.5, E16.5, and P3 were embedded in paraffin, and 6 μm sections were cut by the microtome. Locked nucleic acid (LNA)-modified oligonucleotide probes specific for miR-182 and a negative control containing a single-base mismatch were labeled at the 3′ end with digoxigenin and used for the hybridization. The hybridized probes were detected using anti-digoxigenin antibodies conjugated to alkaline phosphatase (Roche, Mannheim, Germany) and nitroblue tetrazolium/5-bromo-4-chloro-3-indolyl phosphate (NBT/BCIP) was used as the substrate. Three washing temperatures (25 °C, 35 °C, and 45 °C) were tested in the experiments. The sections were counterstained with Kernechtrot stain solution (Muto Chemical, Tokyo, Japan).

### Gene targeting and generation of miR-182 KO mice

An 11.9 kb DNA fragment encoding the hairpin precursor of miR-182 was obtained from the murine 129 SvEv genomic library and subcloned into a 2.4 kb backbone vector. Then, the 75 bp hairpin precursor in the fragment was replaced with the neomycin resistance gene under the control of the phosphoglycerate kinase 1 promoter flanked by loxP/FRT sites. The orientation of the neomycin resistance gene was opposite to that of the hairpin precursor. The resulting targeting construct contained short (2.4 kb) and long (9.4 kb) homology arms on the 5′ and 3′ sides of the neomycin resistance gene, respectively.

The targeting vector was linearized using NotI and transfected into iTL1 129/SvEv embryonic stem (ES) cells via electroporation. After selection in G418 antibiotic, surviving clones were expanded for PCR analysis with primers F2 (Table 1) to identify recombinant ES clones. Positive clones were expanded again and confirmed using PCRs with primers F1 and A3. Of note, the F1 and F2 primers bind to the PGK-Neo-loxP/FRT cassette, whereas the A3 primer is designed to recognize a region outside the short homology arm that is not present in the targeting vector but is found in the murine genome.

**Table 1:** Primer and probe sequences.

**Primer/probe**	**Sequence (5′-3′)**
F1	CGTTCTTCGGACGCCTCGTCAACAC
F2	GGATCCGTTCTTCGGACGCCTCGTC
A3	TCAGAAGCTATACGGCACAGCCAG
108–03K-F	GGACCATACAGGCCGAAGGAC
Neo-R1	CCTTCTATCGCCTTCTTGACGAGTTC
182-R4	CCCAAGTCCTTTTCACCGAGAAGAG
miR182LNA	tgTgaGttCtaCcaTtgCcaAa
miR182LNA/MM	tgTgaGttCtaCca**A**tgCcaAa

Primers used for genotyping the miR-182 KO mice are shown. The perfect-matched LNA-probe against miR-182 (miR182LNA) or the single-base mismatched control probe (miR182LNA/MM) was used for in situ hybridization. LNA positions are in capital letters, with DNA in lower case and the position of the single-base substitution in bold print.

The hemizygous clones were injected into C57BL6/J (B6) blastocysts to generate male chimeras. Heterozygous mice were obtained by backcrossing 129/B6 background mice to wild-type B6 mice for five generations. To genotype the mice, we isolated genomic DNA from the tail and performed PCR analysis using primers 182–03K-F and Neo-R1 or 182–03K-F and 182-R4.

### Immunohistochemistry

Eyes from 12-week-old and 16-week-old mice were enucleated, fixed, sectioned, and immunolabeled as described previously [17]. Briefly, enucleated eyes were fixed in Super Fix (Kurabo, Osaka, Japan) overnight and cryosectioned using standard protocols. The primary antibodies used in this study included 1:600 rabbit anti-pax6 (paired box gene 6) (Covance, Princeton, NJ), 1:1,000 mouse anti-recoverin (Chemicon, Temecula, CA), 1:1,000 mouse anti-glutamine synthetase (GS; Chemicon), 1:2,000 mouse anti-rhodopsin (Ret-P1; Sigma, St. Louis, MI), 1:1,000 rabbit anti-protein kinase Cα (PKCα; Sigma), and 1:1,000 mouse anti-nestin (BD PharMingen, San Jose, CA). The secondary antibodies included anti-mouse or anti-rabbit IgG conjugated to 1:300 FITC (Jackson ImmunoResearch, West Grove, PA). Cell nuclei were stained with 1 µg/ml 4',6-diamidino-2-phenylindole (DAPI; Invitrogen). Each specimen was imaged using a laser-scanning confocal microscope (Leica, Wetzlar, Germany).

### cDNA microarray

Gene expression profiling was performed using a mouse genome 430.2 microarray chip (Affymetrix, Santa Clara, CA) and the effects of the *mir-182* null mutation on target genes were analyzed essentially as previously described by Rodriguez et al. [14], Giraldez et al. [18], and Cheng and Li [19]. In brief, total RNA was isolated from the eyes of 4-week-old wild-type or KO mice using TRIzol reagent (Invitrogen). Hybridization was performed based on the chip manufacturer’s protocol. After first-strand cDNA was synthesized using a T7-Oligo(dT) primer and reverse transcriptase, second-strand cDNA synthesis was performed using DNA polymerase and RNaseH. Biotin-labeled cRNA was synthesized via in vitro transcription using T7 RNA polymerase. Fragmented cRNA was then hybridized to the mouse array and visualized using fluorescent phycoerythrin-conjugated streptavidin. Finally, chips were scanned using a GeneArray scanner (Affymetrix) and analyzed using GCOS software (Affymetrix).

### Data analysis and miR-182 target searching

To examine the effects of the *mir-182* null mutation on the expression of miR-182 target genes, we analyzed cDNA microarray data using a protocol described by Rodriguez et al. [14] and Giraldez et al. [18]. Briefly, after normalization and standardization of the data using Z score transformations [20,21], hybridization intensities from KO and wild-type mice were compared. The differences were expressed as Z ratios and ranked in five groups. It should be noted that the Z ratios were obtained by subtracting the intensities observed for the wild-type mice from those observed for the KO mice. Hence, large Z-ratio values represented higher expression levels in the KO mice. To determine the degree of enrichment for genes with high Z-score values, we calculated the average frequency of sequences complementary to miR-182 in each group. In brief, 3′-UTR sequences from 8,414 genes were obtained from the PACdb database [22]. Sequences complementary to the miR-182 5′-seed sites [6,23] were examined in the 3′-UTR sequences. In this study, we used sequences with four different lengths as those complementary to the 5′ to 3′ sequence of the miR-182 seed sequence: n2–6 (GCCAA), n2–7 (TGCCAA), n1–7 (TGCCAAA), and n1–8 (TTGCCAAA). For comparison, the fold enrichment of the average frequency of sequences targeted by miR-182 was calculated in each group.

Additionally, we analyzed the effective regulatory activities of 398 mouse miRNAs by integrating the cDNA microarray expression data with miRNA target predictions [19]. For this analysis, the predictive values of miRNA targets were first calculated using the miRanda algorithm [24,25] and a total of 5,389,318 miRanda values were obtained for 398 miRNAs and 13,541 mouse genes. Then, cDNA microarray data was normalized using GCOS software (Affymetrix) and the log(2) ratios were calculated with the intensity ratios (WT versus KO). By incorporating all the miRnada values and the log(2) ratios of genes into the WinMIR program kindly provided by Drs. Chao Cheng and Lei M. Li of University of Southern California, the scores of regulatory activity changes (AC scores) of 398 miRNAs were calculated and sorted. It should be noted that a positive AC score indicated enhanced activity from the corresponding miRNA, whereas a negative AC score indicated a deduction in the activity.

## Results

### MiR-182 is highly expressed in the mouse eye

**Figure 1:**
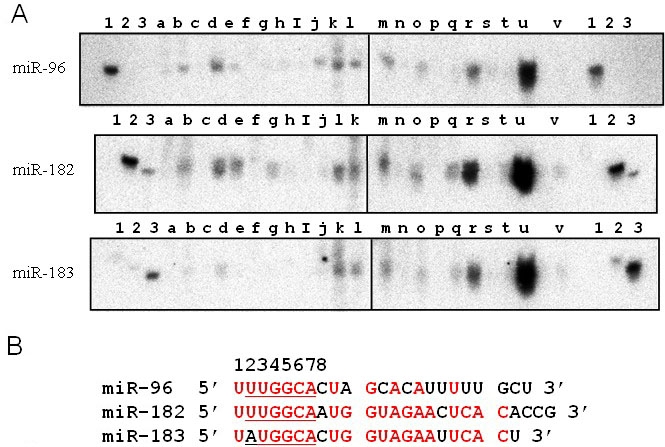
Tissue distribution of miR-96, miR-182, and miR-183 in the mouse. Expression levels of miR-96, miR-182, and miR-183 in lymph node (lane a), skin (b), skeletal muscle (c), white fat (d), brown fat (e), liver (f), kidney (g), adrenal gland (h), spleen (i), testis (j), stomach (k), small intestine (l), large intestine (m), thymus (n), lung (o), cardiac ventricle (p), thyroid gland (q), submandibular gland (r), cerebrum (s), cerebellum (t), and eye (u) tissues of wild-type mice were analyzed by northern blotting. Expression levels of miR-182 in fibroblasts isolated from normal skin were also analyzed (v). Synthetic miR-96 (lane 1), miR-182 (lane 2), and miR-183 (lane 3) were used as controls. **B:** Sequences of miR-96, miR-182, and miR-183 are shown. Nucleotides that are identical between miR-182 and miR-96, and miR-182 and miR-183 are denoted in red. Numbers above the sequences indicate the nucleotide position from the 5′ end. The underlined sequences represent the positions of the 2–7 seed region.

We examined the tissue distribution of miR-96, miR-182, and miR-183 in normal adult mice (Figure 1A). All three miRNAs were strongly expressed in eyes and submandibular glands. These results support the notion that miR-182 is eye-specific [9,26]. It should be noted that miR-96, miR-182, and miR-183 have similar sequences in their 5′ seed regions [6,23] and that 18 of 22 nucleotides in miR-183 were identical to those in miR-182 (Figure 1B). Synthesized miR-96, miR-182, and miR-183 were used as controls for northern blotting (lanes 1–3 in Figure 1A). The specificity of the probe for miR-96 was verified. Although probes for miR-182 and miR-183 cross-reacted, they were distinguishable from each other because of differences in their sizes and signal intensities; miR-183 (22 mer) was shorter than miR-182 (25 mer), and the signal intensities from the cross-reaction were weaker than the specific signal intensities.

**Figure 2:**
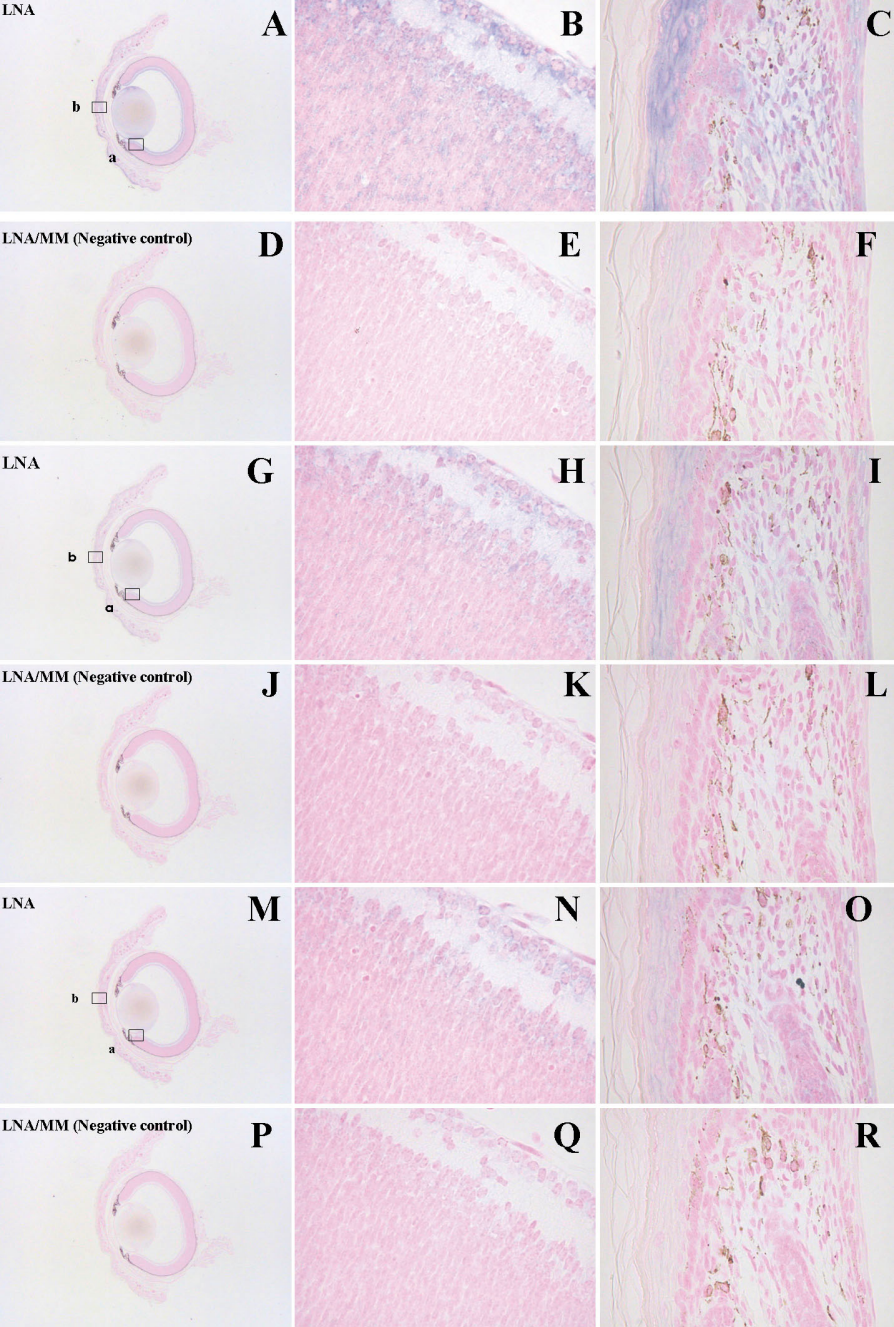
In situ hybridization analysis of miR-182 expression in P3 eyes. A matched LNA probe specific for miR-182 (LNA) or a control probe containing a single-base mismatch (LNA/MM) was used for the hybridization. The probe sequences are shown. The unhybridized LNA probes were washed out at 25 °C (**A-F**), 35 °C (**G-L**), or 45 °C (**M-R**). Magnifications are either ×16 (**A**, **D, G, J, M, P**) or ×400 (**B, C, E, F, H, I, K, L, N, O, Q, R**). The strongest staining was observed at 25 °C and the signals became fainter and eventually disappeared as the washing temperature increased.

**Figure 3:**
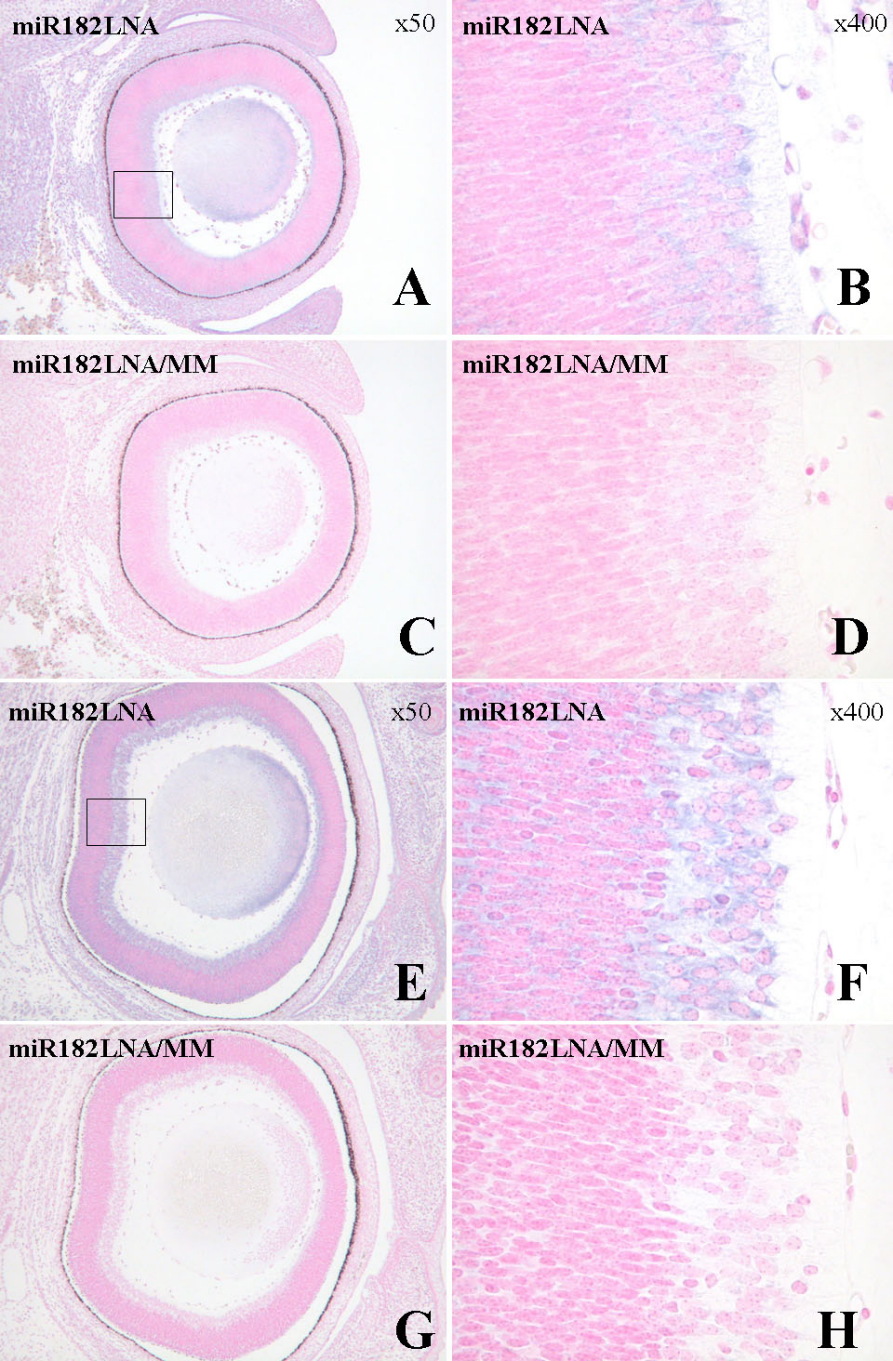
In situ hybridization analysis of miR-182 in eyes of normal embryonic mice. Eyes of embryonic mice on E14.5 (**A-D**) and E16.5 (**E-H**) were analyzed. Matched LNA probes specific for miR-182 (miR182LNA, upper panels) or a control probe containing a single-base mismatch (miR182LNA/MM, lower panels) was used for the hybridization. The unhybridized LNA probes were washed out at 25 °C Note that the matched probe produced the strongest signals in the inner retina (magnified upper panels).

To determine the role of miR-182 during eye development, we identified the specific cells that express miR-182. We performed in situ hybridization analysis in postnatal eyes at P3 (Figure 2), and in embryonic eyes at E14.5 and E16.5 (Figure 3). As shown in Figure 2, strong expression was observed in P3 eye sections obtained from throughout the inner retina, with particularly strong signals detected in the ganglion cells. Yet in the E14.5 and E16.5 eyes, strong signals were observed in the innermost cell layers (Figure 3). These results are consistent because the inner cell layer of embryonic eyes contains progenitor cells differentiating into ganglion cells. The signals in the E16.5 eye were stronger than those in the E14.5 eye, which agrees with a previous report [9]. The specificity of the LNA probe was evaluated using a probe harboring a single-base substitution.

### Generation of miR-182–deficient mice

**Figure 4:**
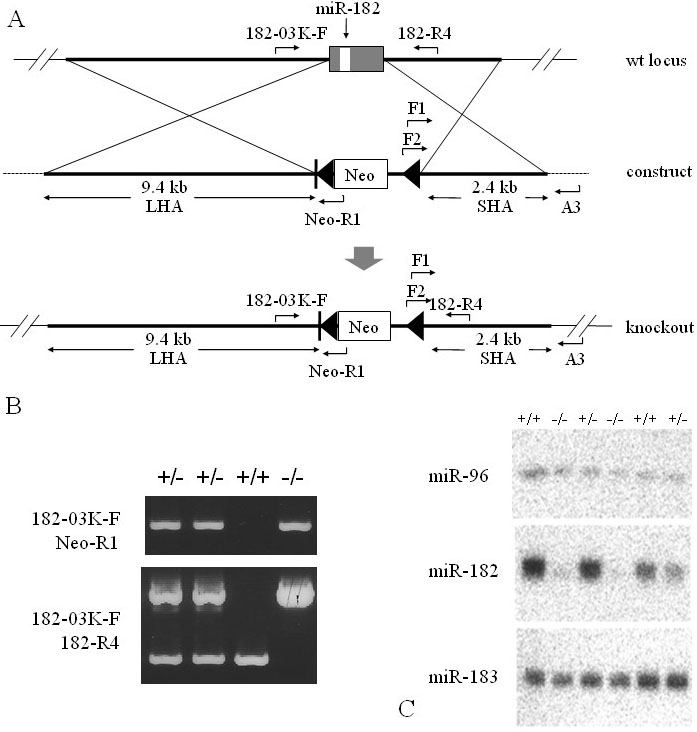
A gene targeting strategy used to generate miR-182 KO mice. **A:** The pre-miR-182 locus was replaced with the Neo cassette flanked by LoxP sites (black triangles). Primers used for genotyping are shown. **B:** Genotyping the miR-182 KO mice. Genomic DNA isolated from wild-type (+/+), heterozygous (+/−), or homozygous KO (−/−) mice was analyzed by PCR using the primers 183–03K-F and Neo-R1 or 183–03K-F and 182-R4. **C:** Mature miR-182 is indeed absent in the eyes of KO mice. Total RNA was isolated from eyes of wild-type (+/+), heterozygous (+/−), and homozygous (−/−) KO mice and the expression levels of miR-96, miR-182, and miR-183 were analyzed by northern blotting. Abbreviations: long homology arm (LHA); short homology arm (SHA); neomycin resistance gene (Neo).

To further examine the biologic role of miR-182 during the retinal development, we generated a miR-182 KO mouse line. Figure 4A depicts a brief description of the targeting strategy, and Figure 4B shows an example of PCR genotyping. Because the precursor sequence of miR-182 was only 75 bp, the entire gene was replaced by the PGK-Neo cassette in the targeting construct.

The homozygous and heterozygous mice were born normally. It appeared that they were morphologically wild type and exhibited no aberrant phenotypes. Northern blot analysis revealed that mature miR-182 was not expressed in the eyes of homozygous KO mice (Figure 4C). The expression patterns of miR-96 and miR-183, encoded as precursor genes in the long homology arm of the targeting construct, were not affected by the deletion.

### Retinal structure in the KO mice

**Figure 5:**
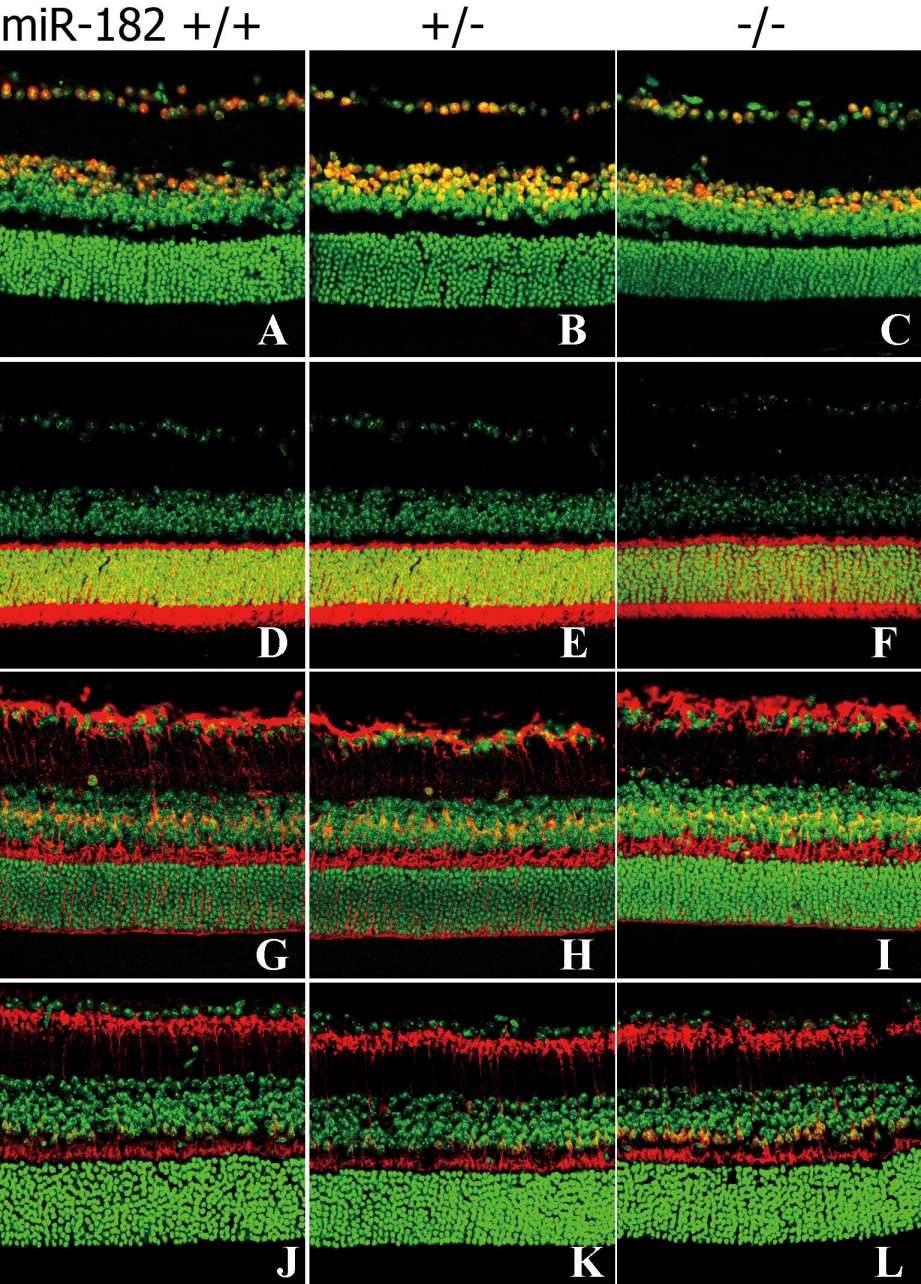
Characterization of cells in retinas from 12-week-old miR-182 KO mice. **A-C:** Ganglion and amacrine cells were labeled using anti-Pax6 antibodies in wild-type (+/+), heterozygous (+/−) and homozygous (−/−) KO mice. **D-F:** Photoreceptor cells were labeled using anti-recoverin antibodies. **G-I:** Müller cells were labeled using anti-GS antibodies. **J-L:** Rod bipolar cells were labeled using anti-PKCα antibodies. Primary antibody labeling is depicted in red. Lower panels show merged labeling patterns with DAPI (green).

**Figure 6:**
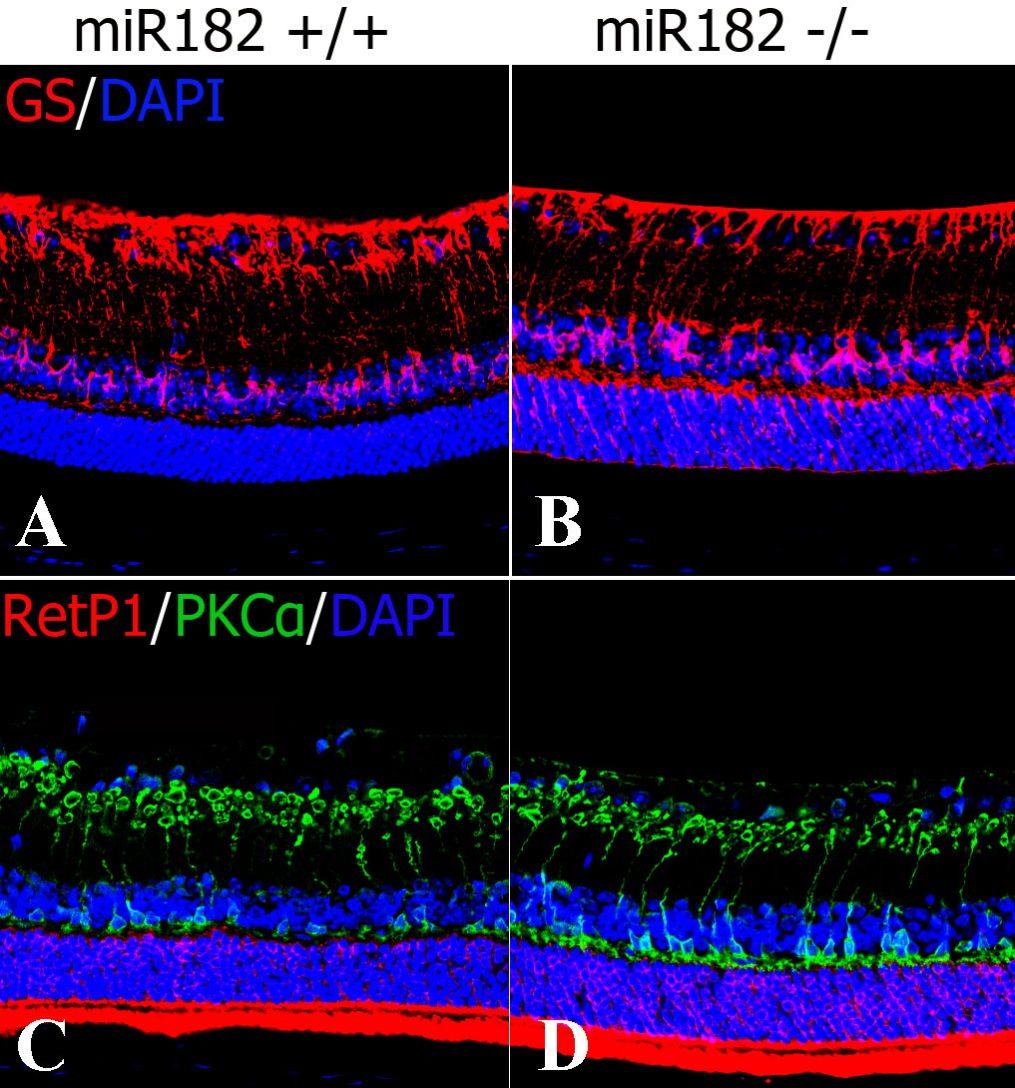
Immunohistochemistry of the 16-week-old retinas. **A, B:** Müller cells in both the wild-type and KO retinas were labeled with anti-glutamine synthetase (GS) antibody. **C,D:** Rod photoreceptors and rod bipolar cells were labeled with anti-RetP1 and anti-PKCα antibodies.

To evaluate the effect of the *mir-182* null mutation on retinal development, we investigated retinal cell populations and layering in 12-week-old and 16-week-old wild-type, heterozygous, and homozygous mutant mice. In Figure 5A-C, the ganglion cells (the first layer from the top) and the amacrine cells (the second layer) were visualized using anti-Pax6 antibodies. Cells in the heterozygous and the homozygous KO mice appeared morphologically normal and similar to those of the wild-type mice. The structure of the photoreceptor cell layer was evaluated by labeling the photoreceptor cells with antibodies specific for recoverin (Figure 5D-F) and rhodopsin (Figure 6). Layer thickness values and the numbers of positive cells, however, were similar among the mice. Furthermore, no structural or quantitative changes in the Müller cells (glutamine synthetase; Figure 5G-I, Figure 6), rod bipolar cells (protein kinase C alpha; Figure 5J-L, Figure 6), or neural progenitor cells (anti-nestin; data not shown) were observed in these mice.

### Transcriptional profiling and target analysis

**Figure 7:**
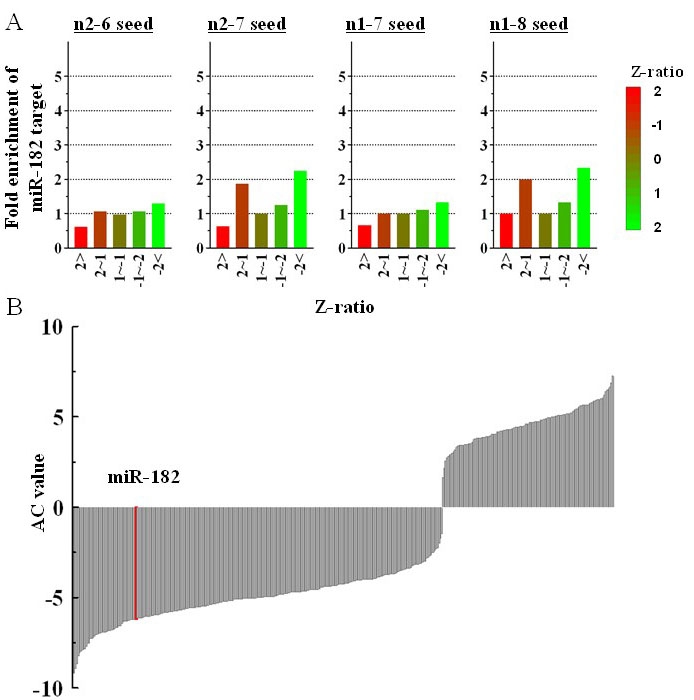
Fluctuation of miR-182 target gene expression in the KO and wild-type mice. **A:** The Z ratios for genes were calculated between the KO and wild-type mice and the genes were classified into five groups. The average frequencies of the 3′ UTR sequences targeted by miR-182 were calculated in each group and the fold enrichment compared with all of the genes was obtained. Four different complementary sequences were used to analyze the potential miR-182 5′ seeds: n2–6 (GCCAA), n2–7 (TGCCAA), n1–7 (TGCCAAA), n1–8 (TTGCCAAA). **B:** The activity change (AC) scores of 398 miRNAs were calculated using the signal log ratios of genes from the wild-type and KO mice. The AC scores of miR-182 (−6.16) is marked with a red bar.

Although miRNAs modulate gene expression post-transcription [27,28], several reports show that the overall expression levels of target mRNAs are specifically reduced by miRNA [14,29-31]. We therefore performed expression profiling of mRNAs in the KO and wild-type mice. The cDNA microarray data was analyzed as described by Giraldez et al. [18] and Rodriguez et al. [14]. The expression ratios (Z ratios) were obtained by subtracting the signal intensities for the wild-type mice from those for the KO mice. Thus, a large Z ratio represented higher expression levels in the KO mice. We reasoned that if the expression levels of the miR-182 target genes were upregulated in the KO mice, the genes with high Z-ratio values would be enriched with miR-182 target sites. As shown in Figure 7A, however, the average frequency of sequences complementary to the miR-182 seed was slightly lower for the genes with higher Z ratios.

Furthermore, we calculated the effective regulatory activities of all miRNAs in the wild-type relative to the KO mice based on the miRanda algorithm [24,25] (see Cheng and Li [19] for further details). As shown in Figure 7B, however, miR-182 was ranked as an inactive miRNA in the wild-type mice relative to the KO mice. These data do not support the notion that miR-182 target gene expression is upregulated in the KO mice.

## Discussion

We focused on miR-182 because this miRNA is robustly expressed in retinas. We then attempted to generate miR-182 KO mice. As an initial report, we focused on the retinal structure of the KO mice. Although recent evidence indicates that miRNAs play key roles in the retina, whether a defect in retina-enriched miRNA in vivo leads to functional loss and subsequent disease is unknown. In this study, we investigated miR-182 expression in both the embryonic and postnatal eyes using miR-182 KO mice.

In line with previous reports [8,9,26], miR-182 was highly expressed in the retina, suggesting it may play important roles in both retinal development and maintenance. Previously, other groups have shown the early developmental and postnatal expression patterns of miR-182 using in situ hybridizations [8-10,26]. These results are inconsistent, however; one study showed signals specifically in the outer retina [8], whereas others have observed signals in both the outer and inner retina [9,10]. Furthermore, Ryan et al. [26] reported that the signals were observed throughout the retinal layers. Our in situ hybridization data obtained using LNA-modified probes showed that miR-182 is strongly expressed in both embryonic and P3 inner retinas, especially in the ganglion cell layer. The discrepant results may be due to the probe specificities as well as different hybridization conditions. It seems that using such short probes makes it difficult to detect the expression pattern precisely.

Damiani et al. [15] demonstrated that retina-conditional Dicer-deficient mice clearly show rosette formation, layer remodeling, and eventually degeneration in the retina. The number of rod bipolar cells was significantly lower in the Dicer KO mice, suggesting miRNAs may be essential for rod bipolar cells [15]. In approximately16-week-old *mir-182* null mutant mice, however, we did not observe any alterations of the retinal organization. Furthermore, we did not observe any significant fluctuation in the expression of target genes in the miR-182–depleted retinas. Thus, miR-182 deficiency alone does not adversely effect normal retinal development or maintenance. This conclusion may be consistent with recent studies showing that miRNAs do not markedly downregulate target gene expression, and, instead, appear to act as rheostats to allow small adjustments to protein output [27,28].

There are several limitations to the present study. First, older animals were not investigated. Retinal defects might develop as the mice age, because older Dicer KO mice showed numerous defects in retinal structures, including cell death [15]. Moreover, the KO mice may have a deficit in their response to light, because the heterozygous Dicer-deficient mice were shown to have such a defect despite a lack of morphological changes [15]. Abnormal responses to damaging light, toxicants, or other stresses also may be present. These possibilities require further examination.

MiR-182, miR-183, and miR-96 have similar seed sequences, target genes, expression patterns, and genomic loci [9], suggesting they may act in a coordinated manner, compensating for each other in vivo. Thus, additional removal of miR-183 as well as miR-96 may be necessary to unravel their exact roles in retinal development.

In addition to its possible roles in the retina, the expression of miR-182 is downregulated during the differentiation of 3T3-L1 pre-adipocytes into adipocytes [32]. Thus, we also hypothesized that miR-182 KO mice could become obese due to increased numbers of adipocytes. The miR-182 KO mice appeared to have no major phenotypes regarding their growth, however. No obvious differences in bodyweight (for example KO male at 7 weeks; 22.5±0.4 g, WT male at 7 weeks; 21.8±0.3 g, N.S. by unpaired Student’s *t*-test) or in the weight of adipose tissues such as epididymal, perirenal, omental, and retroperitoneal adipose tissues were observed so far between the WT and KO mice (unpublished data).

In brief, we successfully generated a miR-182 KO mouse line, which showed no major alterations in their retinal structure. To the best of our knowledge, this is the first loss of function study of a miRNA abundantly expressed in the retina. MiR-182 likely is not a major determinant of retinal development, maintenance, or cell survival. This study is the first step toward elucidating the roles of individual miRNAs in the retina.
